# What is the utility of blood beta-hydroxybutyrate measurements in emergency department in patients without diabetes: a systematic review

**DOI:** 10.1186/s13643-023-02203-7

**Published:** 2023-04-28

**Authors:** Su An Hui, Angela Lin Chiew, Barbara Depczynski

**Affiliations:** 1 National University of Singapore, Singapore, Singapore; 2 Clinical Toxicology & Emergency Medicine Prince of Wales Hospital, Sydney, Australia; 3 Conjoint Associate Professor Prince of Wales Hospital Clinical School, UNSW Medicine, Sydney, Australia; 4 Prince of Wales Hospital, Sydney, Australia

**Keywords:** Ketones, Beta-hydroxybutyrate, Ketosis, Emergency, Hospital

## Abstract

**Background:**

Ketones are synthesised as an alternative fuel source during times of energy restriction. In the absence of a hyperglycemic emergency, ketosis in patients presenting to the emergency department (ED) may indicate reduced carbohydrate intake. In the perioperative setting, excess fasting with ketosis is associated with worse outcomes; however, whether ketosis in patients without diabetes presenting to ED is also associated with worse outcomes is unclear. This systematic review aims to examine the evidence for ketosis in predicting the need for hospital admission in patients without diabetes, presenting to the ED.

**Methods:**

A systematic review was performed using PRISMA guidelines. We searched electronic bases (OVID-Medline, OVID-EMBASE, Scopus and PubMed) up to December 2022. Eligible studies included children or adults without diabetes presenting to the ED where a point-of-care capillary beta-hydroxybutyrate (BHB) was measured and compared to outcomes including the need for admission. Outcome measures included need for admission and length of stay. Content analysis was performed systematically; bias and certainty assessed using standard tools.

**Results:**

The literature search found 17,133 citations, 14,965 papers were subjected to title and abstract screening. The full text of 62 eligible studies were reviewed. Seven articles met the inclusion criteria. Six studies were conducted solely in the paediatric population, and of these, four were limited to children presenting with gastroenteritis symptoms. Median BHB was higher in children requiring hospital admission with an AUC of 0.64–0.65 across two studies. There was a weak correlation between BHB and dehydration score or duration of symptoms. The single study in adults, limited to stroke presentations, observed no relationship between BHB and neurological deficit at presentation. All studies were at risk of bias using the Newcastle-Ottawa Scale and was assessed of “very low” to “low” quality due to their study design in the Grading of Recommendations, Assessment, Development and Evaluation (GRADE) approach. Heterogeneity amongst selected studies precluded meta-analysis.

**Conclusion:**

The evidence for any utility of BHB measurement in the ED in absence of diabetes is limited to the paediatric population, specifically children presenting with symptoms of gastroenteritis. Any role in adults remains unexplored.

**Supplementary Information:**

The online version contains supplementary material available at 10.1186/s13643-023-02203-7.

## Introduction

The ketone bodies β-hydroxybutyrate (BHB), acetoacetate, and acetone are an alternative fuel source primarily utilised by the human body in states of energy restriction with reduced carbohydrate intake, usually characterised by low or normal glucose and appropriately low insulin [1]. BHB is the most abundant ketone body [1]. Normally, circulating BHB levels are < 0.5 mmol/L, but can reach up to 6–7.5 mmol/L during prolonged fasting [2]. Hyperketonemia is defined as BHB > 1.0 mmol/L and ketoacidosis as BHB > 3.0 mmol/L [3, 4].

The most common cause of ketoacidosis amongst patients presenting to ED is diabetic ketoacidosis (DKA), occurring typically in type 1 diabetes, where there is a pathological lack of insulin [5]. More recently, euglycemic ketoacidosis as a complication of sodium-glucose transport protein 2 inhibitors (SGLT2i) has been recognized [6]. Other rarer presentations of ketonaemia or ketoacidosis in adults may occur in association with hyperemesis gravidarum, lactation, or alcoholic ketoacidosis [7, 8].

Children are thought to be more vulnerable to ketosis due to lower glycogen stores and higher metabolic rates compared to adults [4, 9]. Excess fasting perioperatively in young children, or intercurrent illnesses with a period of poor oral intake can result in hypoglycemia and ketosis due to accelerated starvation of childhood (ASC) [10, 11]. However, ketosis in fasted adult patients is uncommon [12]. Many paediatric ED presentations are associated with reduced oral and carbohydrate intake, with one study quantifying 1833 cases of starvation ketosis per 100,000 paediatric presentations (1.8%; 95% confidence interval 1.5–2.2%) [11]. Longer surgical fasting times are associated with greater ketosis and worse hemodynamic parameters [10] and so ketosis in patients presenting to ED may be useful as marker of illness severity or guide the need for hospital admission [12]. BHB measurements via point-of-care (POC) testing is readily available, a survey of 89.9% of UK and Ireland paediatric acute care sites finding it in use, thus examining the utility of such a readily available test is important for patient care [13].

The aim of this study is to conduct a systematic review to assess the utility of ketone measurement, using POC devices measuring BHB, in patients without diabetes presenting to the ED as a predictor of illness severity.

## Methods

### Search strategy and data sources

A systematic review was developed utilising the Preferred Reporting Items for Systematic Review and Meta-Analysis (PRISMA) reporting guidelines and was registered at the International Prospective Register of Systematic Reviews (PROSPERO) with the protocol number CRD42022304390. We searched OVID-MEDLINE, OVID-EMBASE, Scopus, and PubMed from inception until March 2021. The reference lists of relevant articles and existing reviews were also manually searched. The search strategy was formulated using search terms relating 2 main themes: ketones, with keywords “ketone*”, “beta-hydroxybutyrate”, “acetylacetone” and “acetone”, and the emergency department with keywords including “emergency” and “hospital” (see complete search strategy in Additional file 1).

### Study selection/eligibility criteria

#### Study types

Randomised controlled trials, prospective non-randomised cohort studies, retrospective cohort studies and retrospective case-controlled studies with an appropriate comparison group were included. Authors of studies were not contacted for additional unpublished data. Studies were excluded if the patient cohort was less than 6 patients. Review articles, conference abstracts, case reports, articles not published in English, or studies not conducted on humans were excluded.

#### Participants

Eligible studies included patients without diabetes presenting to the emergency department (ED). Studies reporting on both adult and paediatric patients were eligible for inclusion. Studies limited to patients with known diabetes mellitus or who were diagnosed with diabetes mellitus during that presentation were excluded.

#### Intervention

The intervention was point-of-care (POC) BHB measured in the ED.

#### Outcomes

Studies whereby BHB levels were assessed in relation to any objective measure of patient outcomes were included. Clinical outcomes of interest included primarily mortality and mortality scores, secondary outcomes were need for admission to hospital from ED, and any reported indicators of illness severity, such as dehydration scores. Studies were excluded if POC BHB level was not linked to any measurable outcome, and so studies limited to prevalence were also excluded.

### Study selection

All studies retrieved from the four databases were exported to a referencing software (EndNote) with duplicates removed for title and abstract screening. One reviewer screened titles and abstracts for valid articles (S.H.). Following which, two independent reviewers retrieved and screened the full texts for eligibility, using the specified inclusion and exclusion criteria (S.H. and B.D.). Any uncertainty over the study eligibility was resolved through discussions between the two reviewers. If there were any unresolved differences in opinion by these two reviewers, a third reviewer was consulted (A.C.).

### Data collection process

Data extracted included general study characteristics (authors, publication year, country, study design, sample size), patient population (age), details on the intervention (source and collection time of blood sample for ketone measurement), the studies’ primary outcomes, other measured outcomes, and any other key findings.

### Risk of bias and certainty of evidence

Assessment of risk of bias was conducted using the Cochrane-Collaboration risk of bias tool for RCTs and the Newcastle-Ottawa scale for non-randomised studies [14, 15]. Assessment of certainty of evidence of included studies was conducted independently by two authors (S.H. and B.D) using the Grading of Recommendations, Assessment, Development and Evaluation (GRADE) approach [16].

### Data synthesis

Due to the clinical heterogeneity amongst studies, meta-analysis was precluded. Instead, a narrative summary analysis of predefined outcome measures was performed. Additionally, the median and interquartile ranges of ketones from individual studies were compiled quantitatively for each outcome using GraphPad Prism version 9.5.0 for Mac, GraphPad Software, San Diego, CA, USA, www.graphpad.com.

## Results

### Study selection

**Fig. 1:**
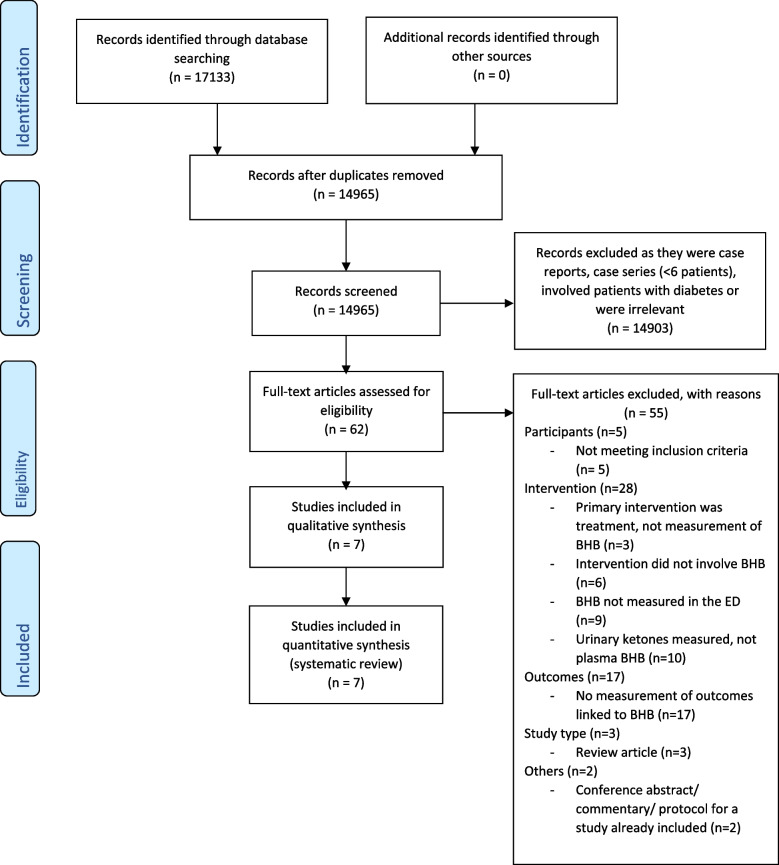
PRISMA flowchart outlining process for inclusion/ exclusion of studies [17]

The initial electronic database search identified 17,133 records, of which 14,965 were unique records (Fig. 1). Of the 62 full-text articles retrieved and assessed for eligibility, 55 were excluded. Reasons for exclusion at full-text review or data extraction stages were participants not meeting the inclusion criteria (*n* = 5), using interventions other than BHB measurements (*n* = 27), lack of measurable outcomes (*n* = 17), study design (*n* = 4), and conference abstract, commentary or protocol for a study already included (*n* = 2) (Fig. 1).

### Study characteristics

The characteristics of the seven included studies are shown in Table 1. Each was undertaken at a single centre. Six reported on paediatric patients only with ages ranging from 0 to 14 years old. Three of the six paediatric studies were limited to children with symptoms of gastroenteritis only [18–20], one was limited to children with benign convulsions with mild gastroenteritis [21], one was limited to children requiring venepuncture as part of their medical work-up [12], and the other paediatric study was limited to children presenting with acute abdominal pain suggestive of paediatric acute appendicitis (PAA) [22]. Only one study was conducted in adults (median age 77 years) and was limited to patients presenting with a diagnosis of first-ever acute stroke [23].

**Table 1:** Characteristics of included studies

Study	Country; sites; recruitment dates	Number analysed; age range (median/mean) at recruitment	Population	Details of intervention	Primary outcome of study	Other outcomes measured
O'Donohoe et al. (2006) [12]	Edinburgh, United Kingdom; single site; November to January	186; 0–154 (median 35.5, IQR 12.0–74.5) months	Convenience sample of patients (< 13 years) presenting to the ED with medical problems requiring blood testing as part of their medical investigations	Venous blood at time of venepuncture BHB using POC MediSense Optium System by Abbott Laboratories, Berkshire, UK	Pattern of BHB levels, Predicting admission vs discharge	Illness severity, Feeding patterns
Durnin et al. (2020) [18]	Dublin, Ireland; single site; April 2016 to February 2017	198; 2 months- 4.99 years (median 1.8 years)	Patients (aged 1 month–5 years) presenting to the paediatric ED with symptoms of vomiting and/or diarrhoea and/or decreased fluid intake with a 4-point Gorelick Scale of 2 or greater or concern at triage of possible hypoglycemia	Capillary blood at triage and 4 h later, BHB using POC Abbott Precision Xceed Pro system (Libertyville, IL, USA)	Relationship between triage POC BHB and the two Gorelick scores	Triage POC BHB and ED admission vs discharge, Triage POC BHB and presenting symptom duration
Levy et al. (2013) [19]	Boston, USA; single site; November 2007 to December 2010	188; median 2.3 years (IQR 1.2–3.5 years)	Patients (aged 6 months–6 years) presenting to the paediatric ED with symptoms of gastroenteritis and were to receive IV fluids for dehydration	Blinded. Venous blood at time of intravenous cannulation. BHB using POC Precision Xtra blood ketone monitoring system (Abbott Laboratories, Libertyville, IL)	Relationship between serum BHB and degree of dehydration, Relationship between serum BHB and degree of metabolic acidosis via serum bicarbonate concentration	Relationship between serum BHB and prospectively assigned appearance score; serum glucose; and duration of symptoms
Pikija et al. (2013) [23]	Varaždin, Croatia; single site; September 1, 2011 to November 30, 2011	51; 31–90 (median 77) years	Adult patients (≥ 18 years) with verified first-ever acute stroke	At presentation capillary blood BHB using POC Precision Xceed Pro β-ketone Monitoring system (Abbott Diabetes Care, Alamedia, CA, USA)	Relationship between capillary BHB and stroke severity (NIHSS score), Relationship between capillary BHB and disability prevalence (mRS score)	-
Torres et al. (2018) [20]	Valladolid, Spain; single site; December 1, 2015 to November 30, 2016	233; 2.9 months to 14.17 years (mean 5.33 years)	Patients (≤ 14 years) who had ≥ 3 vomiting episodes in the last 4 h prior to ED presentation	After initial examination capillary blood BHB (no other details)	Relationship between capillary BHB and failure of oral rehydration	-
Montero et al. (2022) [22]	Navarra; Spain; single site; February to December 2021	151; 2 groups analysed separately-group 1 mean 11.21 years (SD 2.64), group 2 mean 9.87 years (SD 3.08)	Patients (0–14 years) presenting to the paediatric ED with acute abdominal pain suggestive of acute appendicitis with at least 5 days of evolution and association with at least one of the following symptoms: hyporexia, nausea, vomiting, febrile fever, diarrhoea	Capillary blood at time of inclusion in study, on arrival at the paediatric ED, and before initiating intravenous hydration using blood beta-ketone test strip by Abbott Diabetes Care Ltd. analysed by FreeStyle Optimum Neo device	Relationship between capillary BHB and the diagnosis of paediatric acute appendicitis	Number of emetic episodes, days of admission
Lee et al. (2019) [21]	Seoul, South Korea; single site; June 2015 to December 2018	42; mean 21.0 months (SD 11.5 months)	Patients 6 months–6 years of age diagnosed with benign convulsions with mild gastroenteritis	Venous blood BHB (no other information)	Frequency of ketosis in children with benign convulsions with mild gastroenteritis, relationship between ketosis severity and gastroenteritis or seizures	Gastroenteritis symptoms ≥ 4 days, number of seizures before and after ED arrival, and admission to ward

Studies utilised whole blood or capillary blood samples to measure BHB levels and samples were collected at triage, time of venepuncture or after physical examination. Hyperketonaemia was not specifically defined in four of the seven studies [12, 19, 20]. In the two studies [18, 23], hyperketonemia was defined as BHB > 1.0 mmol/L while the one other study [21] defined ketosis and severe ketosis as blood BHB levels of ≥ 0.6 mmol/L and ≥ 4.5 mmol/L respectively.

### BHB levels

**Fig. 2:**
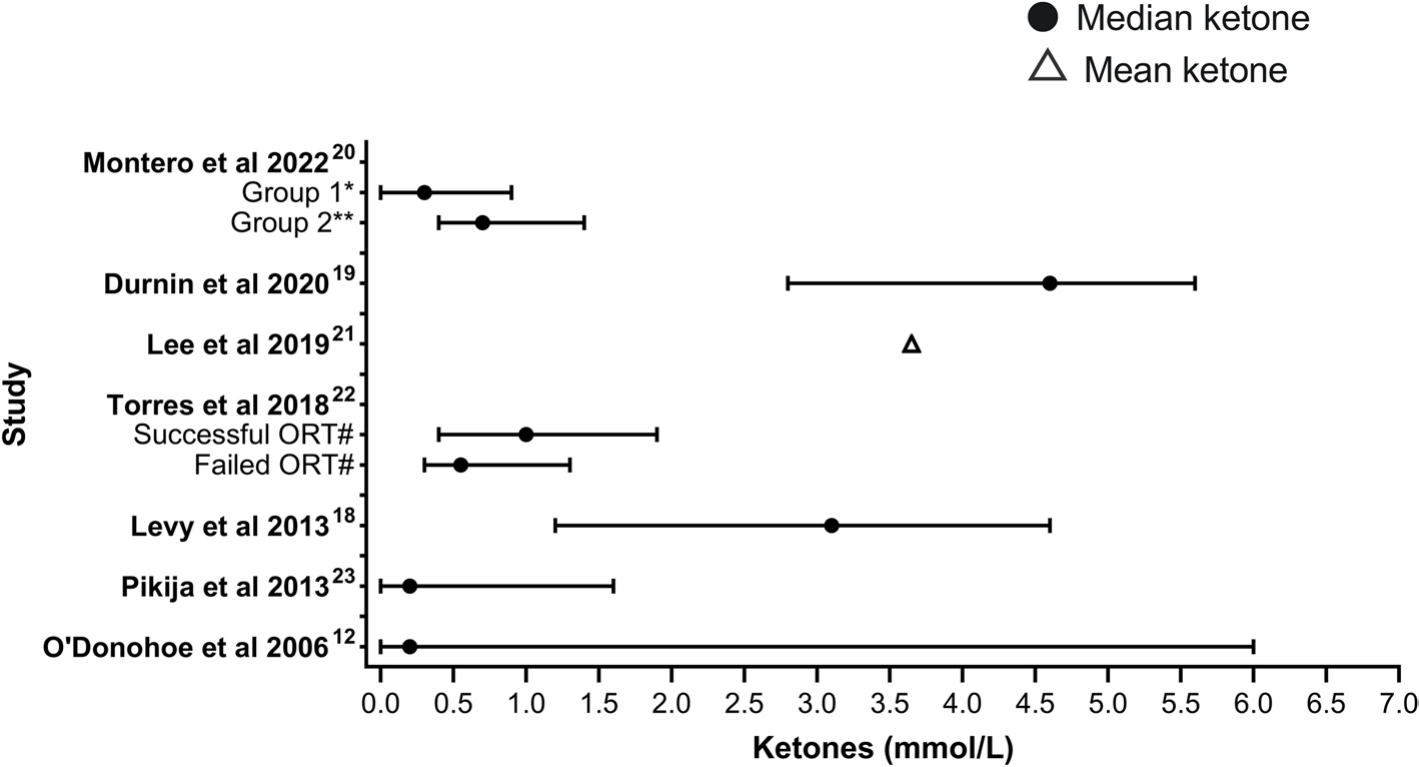
Median/mean BHB with interquartile ranges (IQR) across studies [12, 18–23]. *Group 1: Paediatric patients presenting with non-surgical abdominal pain. **Group 2: Paediatric patients with histologically confirmed acute appendicitis. # ORT: oral rehydration therapy

Median or mean BHB varied across studies. As shown in Fig. 2, numerically higher median or mean BHB were found in the series reported by Levy et al. (2013) (median BHB 3.1 mmol/L), Durnin et al. (2020) (median BHB 4.6 mmol/L) and Lee et al. (2019) (mean BHB 3.65 mmol/L); these studies recruited patients with gastroenteritis symptoms [18, 19, 21]. In contrast, Torres et al. (2018) did not report overall median BHB but reported a numerically lower median BHB in patients presenting with similar symptoms, of 1mmol/L in successful oral replacement therapy (ORT) patients and 0.55 mmol/L in patients who failed ORT, with limited statistical significance *p* = 0.087 [20]. Montero et al.’s (2019) study found median BHB of 0.3 mmol/L in patients with non-surgical abdominal pain, while median BHB in their second group of patients with histologically confirmed diagnosis of acute appendicitis (group 2) was 0.7 mmol/L [22]. Comparatively, Pikija et al. (2013) in adult first-ever stroke presentation and O’Donohoe et al. (2006) in unwell children requiring venepuncture at ED presentation reported lower median BHB levels, both of which were 0.2 mmol/L [12, 23].

### Outcomes

We initially planned to access mortality as a primary outcome, followed by mortality scores and length of hospital stay. However, none of the studies measured these outcomes. As such, admission to hospital versus discharge from ED was selected as the primary outcome of this systematic review. Secondary outcomes included the severity of illness assessed via length of symptoms and dehydration scores upon presentation in studies that recruited patients with likely gastroenteritis. The following sections present narrative summarises for each outcome measure. Table 2 summarises outcomes reported in each study.

**Table 2:** Study results- ketones and measurements of outcomes

**Hospital admission**					
**Study**	**Timing of ketone measurement**	**Admitted median BHB (mmol/L) (IQR)**	**Discharged median BHB (mmol/L) (IQR)**	***P* value**	**ROC curve**
O’Donohoe et al. (2006) [12]	Triage	0.4 (0.3–1.3)	0.2 (0.1–0.7)	*p* < 0.001	AUC 0.64 (95% CI 0.56 to 0.72).
Durnin et al. (2020) [18]	Triage	5.2 (4–6)	4.2 (2.4–5.3)	*p* = 0.001	AUC 0.65 (95% CI 0.57 to 0.73)-optimal cut-off 4.8 mmol/L; sensitivity 61%, specificity 61% for predicting the need for admission
Durnin et al. (2020) [18]	Repeat ketones 4 h later	4.6 (3.3–5.7)	2.9 (1.6–4.2)	*P* < 0.001	AUC = 0.74 (95% CI 0.66 to 0.82)-optimal cut-off 4 mmol/L; sensitivity 64%, specificity 71%
Montero et al. (2022) [22]	During ED visit in patients with suspected acute appendicitis but was ultimately excluded	0.3 (0.1–0.9)	–	–	AUC 0.68 (95% CI 0.53–0.82) (*p* = 0.24), optimal cut-off 0.4 mmol/L; sensitivity 80.70%, specificity 52.17%
Montero et al. (2022) [22]	During ED visit in patients with histologically confirmed acute appendicitis	0.7 (0.4–1.4)	–	–	–
Pikija et al. (2013) [23]	Triage	0.2 (0–1.6 range)	–	–	–
**Dehydration scores**					
**Study**	**Type of dehydration score**	**Median dehydration score (IQR)**	**Median ketones (mmol/L) (IQR)**	**Correlation**	
Durnin et al. (2020) [18]	4-point Gorelick score	2 (2–3)	4.6 (2.8–5.6)	No correlation identified between triage ketones and 4-point Gorelick Scale (Spearman’s ρ = 0.97, p = 0.175)	
Durnin et al. (2020) [18]	10-point Gorelick score	3 (2–5)	4.6 (2.8–5.6)	Weak correlation identified between triage ketones and 10-point Gorelick Scale (Spearman’s ρ = 0.217, *p* = 0.002)	
Levy et al. (2013) [19]	Clinical dehydration score	4 (3–5)	3.1 (1.2–4.6)	Significant positive relationship between serum ketone concentration and the prospectively assigned dehydration score (Spearman’s ρ = 0.22, *p* = 0.003)	
**Symptom duration**					
**Study**	**Median ketones for symptom duration < 1 day (IQR)**	**Median ketones for symptom duration 1–2 days (IQR)**	**Median ketones for symptom duration > 3 days (IQR)**	**Statistical comparisons**	
Durnin et al. (2020) [18]	3.1 (1.1–4.6)	4.3 (2.2–5.7)	4.6 (3.3–5.5)	Statistical significance comparing symptoms < 1 day to symptoms ≥ 1 day (Wilcoxon-Mann-Whitney *U* = 656, *p* = 0.017)	
Levy et al. (2013) [19]	1.3	3.8	4.0	Significant difference in ketone concentrations between symptom duration of < 1 day versus 1–2 days (*p* = 0.001), and between symptom duration < 1 day versus > 3 days (*p* < 0.001), but not between patients with symptom duration 1–2 days versus > 3 days (*p* = 0.736)	

### Admission versus discharge

**Fig. 3:**
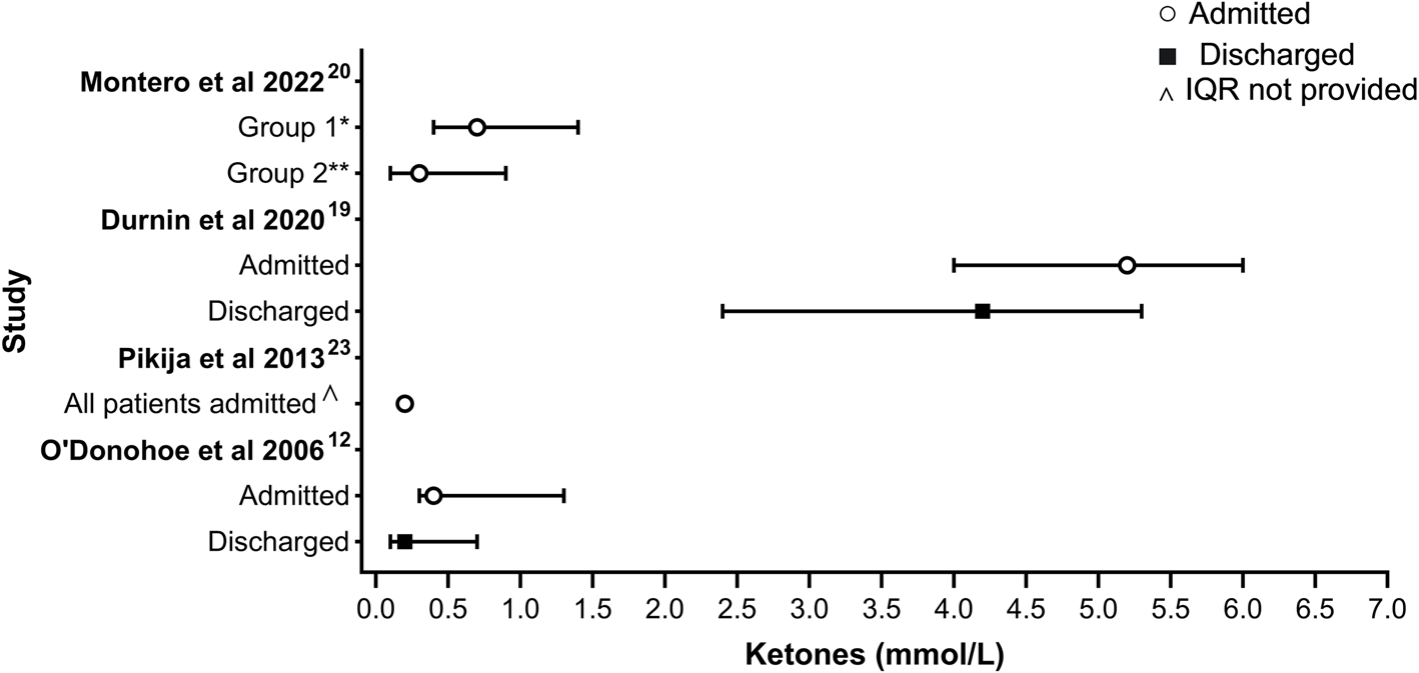
BHB in admitted versus discharged patients [12, 18, 22, 23]. *Group 1: Paediatric patients presenting with non-surgical abdominal pain. **Group 2: Paediatric patients with histologically confirmed acute appendicitis

The BHB values of patients requiring admission as compared to those discharged from ED was reported in 2 studies [12, 18]. All patients were admitted in Montero et al. (2022) and Pikija et al. (2013) with no data on BHB levels of those discharged. Lee et al. (2019) reported admission to ward in 4 patients (26.7%) in the “severe- ketosis” group (BHB ≥ 4.5 mmol/L) compared to 7 patients (25.9%) in the “non-severe ketosis” group (BHB ≥ 0.6 < 4.5 mmol/L) (*p* = 1.000) [22, 23]. The median BHB was higher in admitted patients with gastroenteritis [18], as compared to non-specific medical presentations requiring blood testing as part of their medical work up (Fig. 3) [12]. Data available on the AUC of ROC of triage BHB as a predictor of admission is similar across the two studies- 0.64 (95% CI 0.56 to 0.72) [12] and 0.65 (95% CI 0.57 to 0.73) [18].

### Length of symptoms

**Fig. 4:**
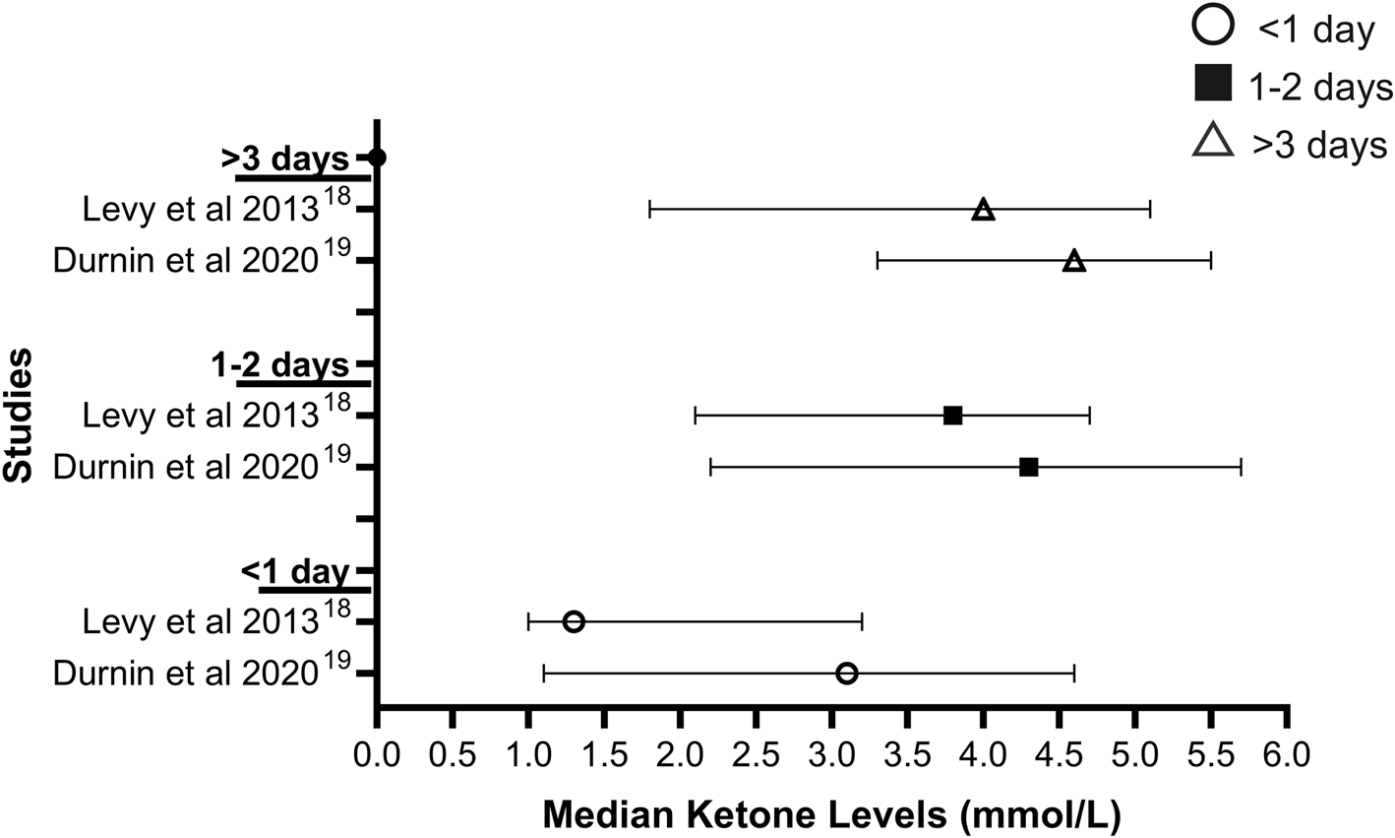
BHB levels according to the length of gastroenteritis symptoms [18, 19]

Two studies, Durnin et al. (2020) and Levy et al. (2013) compared the duration of symptoms of gastroenteritis with ketone concentrations seen in Fig. 4 whereas Lee et al. (2019) reported duration of gastroenteritis symptoms at ED visit ≥ 4 days in 4 patients (36.7%) in the “severe ketosis” group compared to 8 patients (29.6%) in the “non-severe ketosis” group [18, 19, 21]. Both Durnin et al. (2020) and Levy et al. (2013) studied the same time intervals of < 1 day, 1–2 days and > 3 days [18, 19].

The median ketone levels of patients in the groups of different lengths of symptoms < 1 day, 1–2 days and > 3 days, progressively increase within the studies. In Durnin et al. (2020), the association between elevated triage ketones and duration symptoms was noted to be statistically significant comparing symptoms < 1 day to symptoms ≥ 1 day (Wilcoxon-Mann-Whitney *U* = 656, *p* = 0.017) [18]. Levy et al. (2013) reported a statistically significant difference in ketone concentration between patients with symptom duration of < 1 day compared to 1–2 days (*p* = 0.001), and between patients with symptom duration < 1 day compared to > 3 days, *p* < 0.001, but not between patients with symptom duration 1–2 days compared to > 3 days (*p* = 0.736) [19]. However, given that the heterogeneity in the population of patients recruited, whereby with younger median age of 1.8 years in Durnin et al. (2020) compared to Levy et al. (2013) with a median age of 2.3 years, and with Durnin et al. (2020) including patients with symptoms of vomiting and/or diarrhoea and/or decreased fluid intake with 4-point Gorelick Score (4PGS) of 2 or greater or concern at triage of possible hypoglycemia while Levy et al. (2013) recruited children with symptoms of gastroenteritis and were deemed to require IV fluids for dehydration, the two studies cannot be directly compared [18, 19].

### Dehydration scores

Two studies that calculated dehydration scores in children with symptoms of gastroenteritis utilised different dehydration scores, making direct comparison difficult—Durnin et al. (2020) used the 4PGS and 10-point Gorelick Score (10PGS) while Levy et al. (2013) used the Clinical Dehydration Score (CDS) [18, 19]. In Durnin et al. (2020), there was no correlation identified between triage ketone levels and 4PGS (Spearman’s *ρ* = 0.97, *p* = 0.175), and a weak correlation between triage ketones and 10PGS (Spearman’s *ρ* = 0.217, *p* = 0.002) [18]. Levy et al. (2013) identified a positive relationship between serum BHB and the prospectively assigned CDS (Spearman’s *ρ* = 0.22, *p* = 0.003) [19].

### Other markers of illness severity

Several studies measured distinct outcomes which did not allow comparison to the other studies. Within the six studies conducted in the paediatric population, one study measured a single outcome- the failure of oral rehydration and found that initial BHB level had no statistical predictive value of predicting the failure of oral rehydration (*p* = 0.087) [20]. Another distinctive outcome was measured in O’Donohoe et al. (2006) qualitative feeding estimated by parents or caregivers, where it was reported that patients with normal feeding in their cohort had significantly lower median ketones compared to those with less than normal feeding (0.206 mmol/L vs 1.324 mmol/L), with the correlation of BHB to decreased oral intake being *R*^2^ = 0.25 (*p* = 0.001) [12]. Additionally, Levy et al. (2013) explored a general appearance score (1 = obtunded to 5 = alert and active) which was significantly correlated with serum BHB concentrations (*ρ* = − 0.26, *p* < 0.001) [19]. Lee et al. (2019) also quantified patients in the “severe ketosis” compared to the “non-severe-ketosis” groups who had multiple seizures prior to ED arrival and seizure recurrence in the ED as 3 (20.0%) vs 4 (14.8%) patients (*p* = 0.686) and 8 (53.3%) vs 10 (37.0%) patients (*p* = 0.307) respectively [21]. Montero et al. (2022) compared BHB between uncomplicated PAA 0.6 mmol/L (IQR 0.4–0.9) and complicated PAA 1.2 mmol/L (IQR 0.8–1.4) with AUC 0.69 (95% CI 0.54 = 0.85) (*p* = 0.04), and a cut-off point of 1.1 mmol/L, sensitivity 61.1% and specificity 76.9% [22]. Additionally, Montero et al. (2022) correlated other outcomes with BHB, including number of emetic episodes Pearson’s *r* = 0.25 (*p* = 0.03) and days of admission Spearman’s Rho = 0.32 (*p* = 0.02) [22]. Since Pikija et al. (2013) population was specific to stroke, the National Institutes of Health Stroke Scale (NIHSS) was used as a measure of outcome [23]. No relationship was found between admission BHB and NIHSS scores at presentation or day 5 [23].

### Quality appraisal

#### Risk of bias of individual studies

Variability in the risk of bias was minimal according to the Newcastle-Ottawa scale (*n* = 7) whereby all studies included were assessed as cohort studies, including Levy et al. (2013) as it was conducted as a secondary post-hoc analysis of an RCT (Additional file 1) [19].

In terms of representativeness, while O’Donohoe et al.’s (2006) study was not limited to a pre-specified diagnosis like the other studies, it was conducted in the paediatric population requiring venepuncture, thus they were determined to have been conducted in a very selected group [12]. Selection of non-exposed cohort (low ketones) were all drawn from the same community as the exposed cohort and ascertainment of such exposure was all recorded from secure records or structured interviews. Only Lee et al. (2019), a retrospective cohort study had its outcomes present at the start of the study [21]. For outcomes, they were mostly assessed through independent blinding or record linkage and follow-up was assessed as adequate and sufficiently long enough for outcome to occur for all studies.

The main source of variability was comparability. All studies mostly accounted for the variances in population. However, only two studies were explicitly blinded, O’Donohoe et al. (2006), which was blinded to relatives and care providers, and Levy et al. (2013), a secondary analysis of a double-blinded RCT [12, 19].

#### Certainty of evidence

The quality of evidence according to the GRADE system for the included studies (Additional file 1). Most were rated “low” or “very” low, predominantly due to their observational nature.

For admission versus discharge, only O’Donohoe et al. (2006) and Durnin et al. (2020) were assessed as Pikija et al.’s (2013) and Montero et al. (2022)’s population was all admitted, and in Pikija et al.’s (2013), BHB was not a primary outcome measure [12, 18, 22, 23]. Additionally, Lee et al. (2019) presented their data categorically rather than continuously thus proving difficult for comparison [21]. As one study included sample size calculations [18] while the other [12] did not, this outcome was rated “serious” for imprecision and were thus downgraded to “very low”.

For the two secondary outcomes of dehydration scores and length of stay, the same two studies were used—Durnin et al. (2020) and Levy et al. (2013) [18, 19]. Lee et al.’s (2019) study was not included in length of stay due to a difference in length of stay intervals, and the categorical presentation of their data [21]. Since the two studies has similar populations, had direct measures and sample size calculation, inconsistency, indirectness and imprecision were unaffected. However, since the population studies were specific to children with symptoms of gastroenteritis, risk of bias was adversely impacted. Overall, the certainty of evidence was thus rated “low”.

## Discussion

This systemic review found few publications examining the utility of BHB measurement in patients without diabetes presenting to the ED. Most studies were conducted in children. Although the Newcastle-Ottawa Scale revealed no large discrepancy in risk of bias of the non-randomised cohort studies, the GRADE approach downgraded these studies based on their design. The ability of BHB to predict need for admission was modest at best, with optimal cut off levels of BHB of 4 and 4.6 mmol/L [12, 18].

In children with gastroenteritis, a higher median BHB may be linked to a longer duration of symptoms, and so ketosis is more likely to occur in those who have been unable to tolerate oral rehydration prior to presentation [18, 19], however one study found BHB at presentation was not a predictor of failure of ORT in the ED [20]. Similarly, the relationship between BHB levels and dehydration scores may reflect an inability to tolerate oral hydration therapy, otherwise the ingested carbohydrate should have been sufficient to increase insulin/glucagon ratio and switch off BHB production. However, recent animal studies have suggested that glucagon is not the sole primary factor in the regulation of ketone production [24]. Work from an animal model has shown that increased glucocorticoids and catecholamines due to dehydration in the presence of insulinopenia is sufficient to induce ketogenesis [25]. It is unclear whether the gradient of BHB levels from patients with nonsurgical abdominal pain, to uncomplicated PAA to complicated PAA may reflect a greater reduction in carbohydrate intake or illness severity [22]. Whether acute illness with associated increased counter-regulatory hormones coupled with dehydration in a vulnerable patient can induce ketosis and the clinical relevance remains unknown.

Limited data on BHB concentrations in the adult population who present to the emergency department was found. Given the increasing likelihood that of the use of SGLT2i in a broader range of adult patients without diabetes, establishing expected degrees of ketosis with acute illness may be useful in the evaluation of such patients. The single study in the adult cohort was limited to patients presenting with first-ever stroke [23]. BHB is said to have neuroprotective and anti-inflammatory effects [26–28]. In adults, production of BHB is impaired in individuals with COVID-19-induced acute respiratory distress syndrome (ARDS) but not in those with influenza-induced ARDS [29]. Whether finding lower serum concentrations of BHB in patients with newly diagnosed COVID-19 infection presenting to ED might serve as a predictive risk factor for the development of severe COVID-19 remains to be demonstrated [30]. Currently, there is no evidence to support routine BHB testing in adults presenting to the ED.

Studies assessing BHB in other adult populations without diabetes at risk of ketosis were not found. Women presenting with hyperemesis gravidarum may have positive urinary ketones, however it is currently unclear if blood ketones may be more useful in assessing severity and response to therapy, rather than urinary ketones which are slower to clear [31, 32]. While it has been suggested that alcoholic ketoacidosis is a commonly missed diagnosis [33] in ED, the frequency is unclear. Overt ketoacidosis has been reported with low carbohydrate diets [34]. Such presentations to ED is likely to be rare and evident on history as in a series of patients fasting for surgery, increased BHB (> 1 mmol/L) was only seen in 3% of patients [9].

## Limitations

This systematic review is limited by the small number of studies, and a preponderance of observational studies with BHB result being unblinded to the treating team. A high degree of inconsistency/heterogeneity was observed. The reason for heterogeneity is likely due to the paucity of paediatric studies and studies focussing on gastroenteritis. Additionally, some of the studies were conducted using convenience sampling, with potential for selection bias.

When assessing clinical outcome measures, since the majority of studies were conducted in paediatric patients, more appropriate endpoints for such a specific population such as length of symptoms and dehydration scores were reported. However, dehydration scores and length of symptoms are not easily applicable to the rest of the population presenting to the ED without symptoms of gastroenteritis.

## Future directions

Due to the paucity of studies in adults, further studies are required to determine any clinical utility of blood BHB. In this review, several paediatric studies were included, but were mostly a specific subgroup of patients presenting with gastrointestinal symptoms, it is thus unclear whether BHB may have utility in guiding therapy, such as ORT or intravenous rehydration, and whether the change of BHB in response to therapy could be useful for monitoring clinical progress.

## Conclusion

This systematic review reveals limited evidence in the use of ketones in predicting need for hospital admission. The frequency of ketosis in adults without diabetes presenting with acute illness is unclear. Thus, well-designed studies on both the paediatric and adult population without diabetes are required to determine expected degrees of ketosis with acute illness and its clinical utility.

## Supplementary Information

**Additional file 1.** Search strategy. Table 3. Risk of bias using Newcastle-Ottawa Scale. Table 4. Certainty assessment based on outcomes.

## Data Availability

All data relevant to the study are included in the article or uploaded as online supplemental information.
