# Supplementary material for: What is the utility of blood beta-hydroxybutyrate measurements in emergency department in patients without diabetes: a systematic review
Source: Syst Rev. 2023 Apr 28;12:71. doi: 10.1186/s13643-023-02203-7 (PMC10140707; doi:10.1186/s13643-023-02203-7)
Supplement: Supplementary file 1 — Additional file 1. Search strategy. Table 3. Risk of bias using Newcastle-Ottawa Scale. Table 4. Certainty assessment based on outcomes. [file 13643_2023_2203_MOESM1_ESM.docx]

# Appendix

## Search Strategy

### **Ovid MEDLINE(R) ALL <1946 to December 19, 2022>**

1 ketone*.mp. [mp=title, book title, abstract, original title, name of substance word, subject heading word, floating sub-heading word, keyword heading word, organism supplementary concept word, protocol supplementary concept word, rare disease supplementary concept word, unique identifier, synonyms] 55699

2 exp Ketone Bodies/ 11148

3 beta-hydroxybutyrate.mp. or exp 3-Hydroxybutyric Acid/ 9792

4 acetylacetone.mp. 954

5 acetone.mp. or exp Acetone/ 29859

6 emergency department.mp. or Emergency Service, Hospital/ 145874

7 emergency.mp. or exp Emergencies/ 399465

8 traumatic center.mp. 3

9 hospital.mp. or Hospitals/ 1497742

10 Hospitalization/ or acute care.mp. or Critical Care/ 211465

11 1 or 2 or 3 or 4 or 5 93442

12 6 or 7 or 8 or 9 or 10 1839123

13 11 and 12 877

14 limit 13 to (english language and humans) 607

### **EMBASE (until 17 December 2022)**

1 ketone* 75879

2 ‘beta-hydroxybutyrate’ or ‘3 hydroxybutyric acid’ 13947

3 acetylacetone 1853

4 acetone 42931

5 ‘emergency department*’ 207537

6 emergency* 846528

7 ‘acute care’ 51303

8 ‘traumatic center’ 23

9 hospital* 10348873

10 1 or 2 or 3 or 4 128200

11 5 or 6 or 7 or 8 or 9 10661637

12 10 and 11 12079

13 10 and 11 AND [humans]/lim AND [english]/lim 8662

### **PubMed [Until 20 December 2022]**

| **Search number** | **Query** | **Sort By** | **Filters** | **Search Details** | **Results** | **Time** |
| --- | --- | --- | --- | --- | --- | --- |
| **16** | #7 AND #15 |  |  | ("ketones"[MeSH Terms] OR "3-hydroxybutyric acid"[MeSH Terms] OR "acetylacetone"[Supplementary Concept] OR "acetone"[MeSH Terms] OR "ketosis"[MeSH Terms] OR "ketosis"[MeSH Terms]) AND ("emergencies"[MeSH Terms] OR "emergency treatment"[MeSH Terms] OR "emergency medicine"[MeSH Terms] OR "emergency medical services"[MeSH Terms] OR "hospitals"[MeSH Terms] OR "hospitalization"[MeSH Terms] OR "critical care"[MeSH Terms]) | 2,557 | 09:07:50 |
| **15** | #8 OR #9 OR #10 OR #11 OR #12 OR #13 OR #14 |  |  | "emergencies"[MeSH Terms] OR "emergency treatment"[MeSH Terms] OR "emergency medicine"[MeSH Terms] OR "emergency medical services"[MeSH Terms] OR "hospitals"[MeSH Terms] OR "hospitalization"[MeSH Terms] OR "critical care"[MeSH Terms] | 877,463 | 09:07:40 |
| **14** | "critical care" [Mesh] |  |  | "critical care"[MeSH Terms] | 65,383 | 09:07:31 |
| **13** | "hospitalization" [Mesh] |  |  | "hospitalization"[MeSH Terms] | 285,567 | 09:07:24 |
| **12** | "hospitals" [Mesh] |  |  | "hospitals"[MeSH Terms] | 310,889 | 09:07:17 |
| **11** | "emergency medical services" [Mesh] |  |  | "emergency medical services"[MeSH Terms] | 165,311 | 09:07:11 |
| **10** | "emergency medicine" [Mesh] |  |  | "emergency medicine"[MeSH Terms] | 15,396 | 09:07:06 |
| **9** | "emergency treatment" [Mesh] |  |  | "emergency treatment"[MeSH Terms] | 133,142 | 09:07:00 |
| **8** | "emergencies" [Mesh] |  |  | "emergencies"[MeSH Terms] | 42,842 | 09:06:55 |
| **7** | #1 OR #2 OR #3 OR #4 OR #5 OR #6 |  |  | "ketones"[MeSH Terms] OR "3-hydroxybutyric acid"[MeSH Terms] OR "acetylacetone"[Supplementary Concept] OR "acetone"[MeSH Terms] OR "ketosis"[MeSH Terms] OR "ketosis"[MeSH Terms] | 116,748 | 09:06:47 |
| **6** | ketoacidosis [Mesh] |  |  | "ketosis"[MeSH Terms] | 9,760 | 09:06:43 |
| **5** | ketosis [Mesh] |  |  | "ketosis"[MeSH Terms] | 9,760 | 09:06:20 |
| **4** | acetone [Mesh] |  |  | "acetone"[MeSH Terms] | 8,149 | 09:06:15 |
| **3** | "acetylacetone" [Supplementary Concept] |  |  | "acetylacetone"[Supplementary Concept] | 220 | 09:06:08 |
| **2** | "3-hydroxybutyric acid" [Mesh] |  |  | "3-hydroxybutyric acid"[MeSH Terms] | 4,526 | 09:06:01 |
| **1** | ketone [Mesh] |  |  | "ketones"[MeSH Terms] | 108,517 | 09:05:38 |

### **Scopus (until 19 Dec 2022)**

( ( TITLE-ABS-KEY ( ketone* )  OR  TITLE-ABS-KEY ( "beta-hydroxybutyrate" )  OR  TITLE-ABS-KEY ( acetylacetone )  OR  TITLE-ABS-KEY ( acetone )  OR  TITLE-ABS-KEY ( ketosis )  OR  TITLE-ABS-KEY ( ketoacidosis ) ) )  AND  ( ( TITLE-ABS-KEY ( "emergency department" )  OR  TITLE-ABS-KEY ( emergency )  OR  TITLE-ABS-KEY ( "acute care" )  OR  TITLE-ABS-KEY ( "traumatic centre" )  OR  TITLE-ABS-KEY ( hospital ) ) )  AND  ( LIMIT-TO ( EXACTKEYWORD ,  "Human" ) )  AND  ( LIMIT-TO ( LANGUAGE ,  "English" ) )

**5,034**document results

## Risk of bias and certainty of evidence

intriguing as this level did not differ despite the two studies’ population being quite different.

### Risk of bias of individual studies

The risk of bias of the studies were determined using the four criteria summarised in the GRADE handbook for non-randomised observational studies seen in Table 3. Each criteria was judged as “low risk”, “high risk” or “unclear risk”: Upon judging each criteria, the overall risk of bias for the study was rated as “not serious”, “serious”, “very serious” or “extremely serious”.

All studies were assessed as non-randomised cohort studies, including Levy et al. (2013) as it was a secondary post-hoc analysis of an RCT. In terms of representativeness, while O’Donohoe et al.’s (2006) study was not limited to a pre-specified diagnosis like the other studies, it was conducted in the paediatric population requiring venepuncture, thus they were determined to have been conducted in a very selected group. Selection of non-exposed cohort (low ketones) were all drawn from the same community as the exposed cohort and ascertainment of such exposure was all recorded from secure records or structured interviews. Only Lee et al. (2019), a retrospective cohort study had its outcomes present at the start of the study^20^, For outcomes, they were mostly assessed through independent blinding or record linkage and follow-up was assessed as adequate and sufficiently long enough for outcome to occur for all studies.

The main source of variability was comparability. All studies mostly accounted for the variances in population. However, only two studies were explicitly blinded- O’Donohoe et al. (2006), which was blinded to relatives and care providers, and Levy et al. (2013), a secondary analysis of a double-blinded RCT.

| **Study** | | **Selection** | | | | **Comparability** | | **Outcome (illness severity)** | | | **Score** |
| --- | --- | --- | --- | --- | --- | --- | --- | --- | --- | --- | --- |
| Author | Year | Representativeness of exposed cohort | Selection of non-exposed cohort | Ascertainment of exposure | Outcome not previously present | Controls for most important | Controls for other factors | Assessment | Follow-up length | Follow-up adequacy |  |
| O’Donohoe | 2006 | - | **¯** | **¯** | **¯** | **¯** | **¯** | **¯** | **¯** | **¯** | 7 |
| Durnin | 2020 | - | **¯** | **¯** | **¯** | **¯** | - | **¯** | **¯** | **¯** | 7 |
| Levy | 2013 | - | **¯** | **¯** | **¯** | **¯** | **¯** | **¯** | **¯** | **¯** | 8 |
| Pikija | 2013 | - | **¯** | **¯** | **¯** | **¯** | - | **¯** | **¯** | **¯** | 8 |
| Torres | 2017 | - | **¯** | **¯** | **¯** | **¯** | - | **¯** | **¯** | **¯** | 8 |
| Lee | 2019 | - | **¯** | **¯** | **-** | **¯** | **¯** | **¯** | **¯** | **¯** | 7 |
| Montero | 2022 | - | **¯** | **¯** | **¯** | **¯** | **¯** | **¯** | **¯** | **¯** | 8 |

*Table 3: Risk of Bias using Newcastle-Ottawa Scale*

### Risk of bias across studies

The GRADE approach assesses 6 domains- study design, risk of bias (RoB), inconsistency, indirectness, imprecision and other considerations. These domains are assessed across the studies for each outcome, seen in Table 4, with explanations.

*Table 4: Certainty Assessment based on Outcomes*

| **Certainty assessment** | | | | | | | **Certainty** |
| --- | --- | --- | --- | --- | --- | --- | --- |
| **№ of studies** | **Study design** | **Risk of bias** | **Inconsistency** | **Indirectness** | **Imprecision** | **Other considerations** |  |
| **Admission (assessed with: Admission or discharge)** | | | | |  |  |  |
| 2 | observational studies | serious^a^ | not serious | not serious | serious^b^ | all plausible residual confounding would suggest spurious effect, while no effect was observed | ⨁◯◯◯ Very low |
| **Dehydration scores (assessed with: 4-point, 10-point Gorelick Scale, Clinical Dehydration Score)** | | | | |  |  |  |
| 2 | observational studies | serious^c^ | not serious | not serious | not serious | all plausible residual confounding would suggest spurious effect, while no effect was observed | ⨁⨁◯◯ Low |
| **Length of symptoms (assessed with: Days; Scale from: 0 to >3)** | | | | |  |  |  |
| 2 | observational studies | serious^c^ | not serious | not serious | not serious | all plausible residual confounding would suggest spurious effect, while no effect was observed | ⨁⨁◯◯ Low |

**Explanations**

a. The 2 studies were in paediatric patients, one of which was specific to acutely unwell children who had gastroenteritis. One study was blinded to relatives and caregivers while the other was unblinded.

b. While 1 study came close to the required sample size calculated, the other did not mention any such calculations.

c. Both studies were
